# Recent acquisitions in the medical treatment of infertility caused by Chlamydia Trachomatis

**Published:** 2013-06-25

**Authors:** A Al-Moushaly

**Affiliations:** Ghencea Medical Center, Bucharest, Romania; University Emergency Hospital, Bucharest, Romania

**Keywords:** infertility, Chlamydia Trachomatis, genital infection, pregnancy, Azithromycin

## Abstract

The infertility defined as the incapacity of the people to conceive a child in a given period, usually of 1-2 years of sexually unprotected relations, represents a major dysfunction of the genital apparatus. Its frequency is estimated at 10-15% of the couples at the reproductive age. The incidence of sterility is high, a couple out of 10 being sterile. The conjugal sterility is a phenomenon representative for the couple; the woman is responsible for it only in 35-40% of the cases, in 40% of the cases, the male factor is involved. In 20% of the cases, mixed factors are met, both feminine and masculine, and in 5-10% of the cases, the causes cannot be detected. From the multitude of causes of infertility, the infectious factor plays an important role, the Chlamydia infections being lately blamed in the etiology of sterility.

The infections due to Chlamydia Trachomatis (CT) represent the most frequent sexually transmitted diseases, which, most of the times lead to sterility.

Taking into account the widespread of this bacterium in the sexually active population, the effective treatment of the CT infection is very important.

We have selected 200 cases with PID genital infection in the study. All the selected patients had at least 2, 3 and more than 3 inflammation recurrence episodes, this way being considered cases with medium and severe forms of disease. All these selected patients had at least 2, 3 and more than 3 episodes of inflammation recurrence, this way being considered medium and severe disease cases.

In conclusion, there is a high clinical efficiency of the azithromycin treatment in PID case.

## Introduction

The infertility defined as the incapacity of the people to conceive a child in a given period, usually of 1-2 years of sexually unprotected relations, represents a major dysfunction of the genital apparatus. Its frequency is estimated at 10-15% of the couples at the reproductive age. The incidence of sterility is high, a couple out of 10 being sterile. The conjugal sterility is a phenomenon representative for the couple; the woman is responsible for it only in 35-40% of the cases, in 40% of the cases, the male factor is involved. In 20% of the cases, mixed factors are met, both feminine and masculine, and in 5-10% of the cases, the causes cannot be detected. From the multitude of causes of infertility, the infectious factor plays an important role, the Chlamydia infections being lately blamed in the etiology of sterility. 

### Considerations regarding the recent treatment of the Chlamydia Trachomatis of the genital infections

The infections due to Chlamydia Trachomatis (CT) represent the most frequent sexually transmitted diseases, which, most of the times lead to sterility. The World Health Organization estimates that 90 million new cases are produced annually. Many of the pelvic affections, the pelvic inflammatory diseases have Chlamydia Trachomatis as a pathogen agent [**14**]. The teenagers and the young adults are more often affected (one of 10 adolescents is infected; the age group which is the most affected being the one between 15 and 35 years old). 75% of the infected women and 50% of the infected men are asymptomatic, most of them being undiagnosed and untreated [**1,6**]. 

 That is why the CT infection is considered a silent affection [**1,10**]. 

 Taking into account the widespread of this bacterium in the sexually active population, the effective treatment of the CT infection is very important. The old prescription contained tetracycline 500mg/day per os minimum 7 days or doxycycline 200mg/day for 10 days. The problem of these treatments is reflected especially on their use in pregnancy, having an adverse effect on the fetus. The tetracyclines cross the placenta and are found in the fetal tissues, having a toxic potential, mostly on the fetal bone development [**15,17,18**]. 

 However, a rapid and effective treatment is indispensable in the pregnancy because CT determines a series of extremely harmful effects both for the mother and for the fetus. Among all these, the most important are chorioamnionitis, early membrane breaking, intrauterine growth latency and even the pregnancy stopping from progression. Because this bacterium is widespread in the sexually active population, the effective treatment of the Chlamydia infection is very important [**9,16**]. Most of the times, it requires prolonged and repeated courses of treatment, according to how old the symptomatology is. Given the fact that in most of the couples the Chlamydia infection in a partner has as a result the infection of the other partner, the parallel treatment during the same period of time is also recommended for the partner [**7,13**]. 

 Probably, the Chlamydia infection is also responsible for a significant percent of the cases of male infertility, where an old untreated infection is present. 

 200 cases of PID with CT genital infection have been selected and analyzed, on a period of 4 years, from 2003 to 2007, diagnosed according to the clinical manifestations and the infertility state. 

 The purpose of the study was to analyze the main aspects regarding the symptomatology, diagnosis and treatment of the CT genital infection. The main objective of the research is the impact on sterility.

 We have selected 200 cases with PID genital infection in the study. All the selected patients had at least 2, 3 and more than 3 inflammation recurrence episodes, this way being considered cases with medium and severe forms of disease. All these selected patients had at least 2, 3 and more than 3 episodes of inflammation recurrence, this way being considered medium and severe disease cases. The selection of the cases took into account the clinical manifestations, the local examination and the personal pathological history of the patients. This study also tried to evaluate the severity of the PID genital inflammation. 

 The confirmation of the diagnosis was done by highlighting CT in the samples on special media after the insemination of the pathological product taken from the endocol of the patients and serological tests, ELISA test which has determined the presence in the serum of type Ig A, Ig M, Ig G anti chlamydia specific antibodies. 

 Accordingly, out of 200 cases, 100 cases have been classically treated with doxycycline 200mg/day for 10-14 days and the rest of 100 cases have been given azithromycin (Sumamed) 500 mg/day for 3 consecutive days, while repeating the same treatment schemes at 10 days, for a month (3 cures of 3 days per month). 

 Azithromycin (Sumamed) belongs to the macrolides class, the so-called azalides, and, even if they are similar to erythromycin, there are many differences between them as far as the pharmacological and antibacterial activity is concerned. Azithromycin has a higher intracellular concentration, compared to the serum one. The effect is prolonged, its concentrations in the uterine and cervical tissue being higher than the minimum inhibitory concentration for CT, for 20 days or more. Azithromycin does not interact with other medicines and does not have hepatotoxicity [**5,8**], moreover, it can also be used in pregnancy, only one oral dose being enough for the treatment of an uncomplicated chlamydia infection. According to a study done in Louisiana University, it has been proved that the administration of only one single azithromycin dose is enough for the treatment of uncomplicated chlamydiosis, the cure rate being of 98% [**11**].

 The 200 patients have been asked to come back for a reevaluation after 1 and 2 years, the number of pregnancies after the treatment being the core interest. 

 The major purpose of the study was the following of the number (percent) of pregnancies obtained after the treatment. The patients have been evaluated at 1 and 2 years; it was noted that 46 pregnancies (23%) were on term and 10 (5%) were ectopic pregnancies.

**Table 1:** The percent of on term pregnancies and ectopic pregnancies obtained after the PID treatment

Number of treated PID cases	Number of on term pregnancies (%)	Number of ectopic pregnancies (%)
200	46 (23%)	10 (5%)

The high incidence of PID justifies the high percent of ectopic pregnancies, confirming the data in the specialty literature, as P. Vartej (2002) highlights. The inflammatory process interests less the tubal permeability, however, the alteration of the epithelium remains irreversible. Many times, the woman gets pregnant even before the complete healing, due to the antibiotherapy, the inflammatory process interests less the tubal permeability, however, the alteration of the epithelium remains irreversible [**2,3**]. 

 It must be highlighted that the antibiotics used, azithromycin and doxycycline, did not influence the percent of pregnancies, this being the same in the case of both antibiotics. What should be taken into account is the fact that azithromycin can be also used during the pregnancy. This way, the results certify the data in the specialty literature. 

In Sweden, the percent of ectopic pregnancies is of 1,4% in women who have done regular controls, compared with 9,1% in the ones with PID. The risk of appearance of the ectopic pregnancies rises with the number of PID episodes, 33% of the ectopic pregnancies being due to CT. A significant decrease in the appearance incidence of an ectopic pregnancy in women aged 20-24 years has been noticed, if the infection is serious the degree of fertility decreases on long-term and the subsequent episodes have a higher impact on women with a severe disease than in the ones with light forms of the disease [**1,12**]. 

## Conclusion

 The conclusion of these specialty studies, as well as the one done in our clinic shows the high clinical efficiency of the azithromycin treatment in PID case. 

The very good tolerance and the high compliance of the azithromycin treatment makes it the first choice of treatment in the CT infection, bacteriologically confirmed [4,6].

 The efficiency of the genital chlamydiosis treatment with azithromycin is 10% higher compared with the treatment with doxycycline. The therapy compliance is of 100%, the adverse reactions are scarce and minor, azithromycin having the advantage of being administered in pregnancies. 

 After the azithromycin treatment, a number of 46 on term pregnancies (23%) and 10 ectopic pregnancies (5%) have been obtained; the high percent of ectopic pregnancies being explained by the fact that the woman can get pregnant even before the complete cure. 
